# Migration arrest of chemoresistant leukemia cells mediated by MRTF-SRF pathway

**DOI:** 10.1186/s41232-020-00127-6

**Published:** 2020-07-06

**Authors:** Maho Morimatsu, Erika Yamashita, Shigeto Seno, Takao Sudo, Junichi Kikuta, Hiroki Mizuno, Daisuke Okuzaki, Daisuke Motooka, Masaru Ishii

**Affiliations:** 1 Department of Immunology and Cell Biology, Graduate School of Medicine and Frontier Biosciences, Osaka University, Osaka, Japan; 2 WPI-Immunology Frontier Research Center, Osaka University, Osaka, Japan; 3 Laboratory of Bioimaging and Drug Discovery, National Institutes of Biomedical Innovation, Health and Nutrition, Osaka, Japan; 4 Department of Bioinformatic Engineering, Graduate School of Information Science and Technology, Osaka University, Osaka, Japan; 5 Genome Information Research Center, Research Institute for Microbial Diseases, Osaka University, Osaka, Japan

**Keywords:** In vivo imaging, Microscopic imaging, Leukemia, Bone marrow, Cell migration, Cell motility, Chemoresistance, Cancer therapy, Serum response factor (SRF)

## Abstract

**Background:**

Dormant chemotherapy-resistant leukemia cells can survive for an extended period before relapse. Nevertheless, the mechanisms underlying the development of chemoresistance in vivo remain unclear.

**Methods:**

Using intravital bone imaging, we characterized the behavior of murine acute myeloid leukemia (AML) cells (C1498) in the bone marrow before and after chemotherapy with cytarabine.

**Results:**

Proliferative C1498 cells exhibited high motility in the bone marrow. Cytarabine treatment impaired the motility of residual C1498 cells. However, C1498 cells regained their migration potential after relapse. RNA sequencing revealed that cytarabine treatment promoted MRTF-SRF pathway activation. MRTF inhibition using CCG-203971 augmented the anti-tumor effects of chemotherapy in our AML mouse model, as well as suppressed the migration of chemoresistant C1498 cells.

**Conclusions:**

These results provide novel insight into the role of cell migration arrest on the development of chemoresistance in AML, as well as provide a strong rationale for the modulation of cellular motility as a therapeutic target for refractory AML.

## Background

Chemotherapy with metabolic antagonists, such as cytarabine (cytosine arabinoside), has been widely used for the treatment of acute myeloid leukemia (AML) for more than 40 years [1–3]. Nevertheless, even high doses of cytarabine result in disease remission in only 40% of AML patients due to the development of chemoresistance [4, 5]. Chemoresistant AML cells interact with various cell types in the bone marrow (BM), including endothelial cells, osteoblasts, and stromal cells, and these interactions are believed to play essential roles in the development of resistance [6]. Furthermore, compared to T cell acute leukemia cells, AML cells interacted extensively with the BM microenvironment in an MLL-AF9-driven AML mouse model [7, 8]. In vivo studies led to the identification of several candidate molecules with important roles in the interaction between AML cells and BM microenvironment components [9–13].

On the other hand, another study suggested that AML cell migration did not depend on interactions with ligands found in the BM stroma [7]. These contradictive findings highlight the complexity of the mechanisms underlying the interactions between AML cells and BM microenvironment components. The identification of the key BM components that regulate AML chemoresistance remains a significant unmet need in the treatment of AML. Moreover, characterization of the exact localization and movements of chemotherapy-resistant AML cells within the BM is of high importance for a better understanding of the mechanisms underlying resistance.

Herein, we provide further insight into the behavior of chemoresistant AML cells in vivo with evidence from intravital time-lapse bone imaging. We further identify the key molecular mechanisms of migration arrest and chemoresistance development in AML cells.

## Methods

### Mice

C57BL/6 J mice were purchased from Crea Japan Inc. (Tokyo, Japan). Female mice (8–10 weeks of age) were used unless stated otherwise. The number of animals used per group in each experiment is provided in the corresponding figure legend. All mice were maintained under specific pathogen-free conditions, and the Institutional Animal Care and Use Committee of Osaka University approved all procedures involving animals.

### Cells

C1498 cells were purchased from American Type Culture Collection. The green fluorescent protein (GFP) coding sequence was cloned in a pMYs retroviral vector (Cell Biolabs, Inc.). The recombinant virus was produced using the Platinum-E retroviral packaging cell line, Ecotropic (Cell Biolabs, Inc.), which was transfected using PEI MAX (Polysciences, Inc.). C1498 cells were stably transduced using the recombinant retrovirus, grown in DMEM (no glucose or sodium pyruvate, with l-glutamine; Nakalai Tesque, Inc.) supplemented with 10% fetal bovine serum (FBS). Successfully transduced cells were selected by cell sorting and expanded as single-cell clones. One clone was selected for each transfected cell line (C1498-EGFP). C1498-EGFP cells were maintained in DMEM supplemented with 10% FBS and 1% penicillin-streptomycin at 37 °C in a humidified atmosphere containing 5% CO_2_.

### Animal models

The AML syngeneic mouse model was generated by intravenously injecting 2.0 × 10^6^ C1498-EGFP cells resuspended in 100 μL of phosphate-buffered saline (PBS) in C57BL/6 J mice.

### Treatment of mice

AML mice were treated with high-dose cytarabine chemotherapy. Twenty micrograms of cytarabine (Tokyo Chemical Industry Co., Ltd.) dissolved in 200 μL PBS was injected intraperitoneally at days 19 and 20 after C1498-EGFP cell injection for intravital imaging; for survival analysis, chemotherapy was administered at days 14 and 15. In addition to cytarabine, some mice also received CCG-203971. For imaging, 200 μg of CCG-203971 dissolved in 50 μL DMSO solution was intraperitoneally injected at day 18, and twice per day at days 19 and 20. For survival curve analysis, 200 μg of CCG dissolved in 50 μL DMSO was administered at day 13, and twice per day at days 14 and 15.

### Intravital imaging

Intravital two-photon imaging was performed as previously described [14–17]. Imaging was performed using a Nikon upright two-photon microscope (A1RMP) and a laser (Chameleon Vision II Ti: Sapphire; Coherent) at 880 nm; for imaging, a ×25 water immersion objective (APO, N.A. 1.1; Nikon, Tokyo, Japan) was used. Raw images were processed to construct 3D images and maximum intensity projections (MIP) using the Imaris software v9.2.1 (Bitplane). Detection of bone tissues was achieved by second harmonic generation (SHG). And blood vessels were visualized by intravenously injecting Isolectin GS-IB4 from *Griffonia simplicifolia*, Alexa Fluor® 594 conjugate (50 μg/mL; Thermo Fisher).

### Image quantification

The distance between the AML cells and the bone or blood vessels was analyzed using the Imaris software. AML cells were defined as spots with a diameter of 10 μm using the 3D images. The surface of the bones and blood vessels was identified in the same 3D image and transformed to distance gradients. Subsequently, the intensities of the distance gradients of the spots were calculated. We analyzed the displacement area of AML cells by calculating IoUs (intersection over union). Initially, all frames (green channels) of MIP images from each experiment were combined, and thresholds were calculated by the Otsu method. These thresholds were used to distinguish cellular regions from the background. Denoising of the resulting binary images was performed by applying median filters. Finally, we calculated the IoUs between the cell regions of successive frames. To calculate the mean migration speed, we defined AML cells as estimated spots with a diameter of 10 μm from the MIP image; the spot trajectories were automatically analyzed.

### Sorting AML cells

First bone marrow cells were collected from femur, tibia, and pelvis from three untreated and three cytarabine-treated mice 21 days after AML cell injection. The bone marrow cells were blocked with anti-CD16/32 antibody (553141; BD Biosciences) for 5 min and stained with PE conjugated anti-CD49b antibody (553858; BD Biosciences) for 30 min. GFP^+^ CD49b^+^ cells were isolated as AML cells using an SH800 cell sorter (Sony).

### RNA-Seq

Total RNA was extracted using QIAzol lysis reagent (Qiagen) according to the manufacturer’s instructions. Sequencing was performed on an Illumina HiSeq 2500 platform (Illumina) in a 75-base single-end mode and the Illumina Casava software (v1.8.2; Illumina) was used for base calling. Sequenced reads were mapped to mouse reference genome sequences (mm10) using TopHat (v2.0.13) in combination with Bowtie2 (v2.2.3) and SAMtools (v0.1.19). The fragments per kilobase of exon per million mapped fragments were calculated using Cufflinks (v2.2.1). Upstream regulator analysis and causal network analysis was performed using Ingenuity Pathway Analysis (IPA; Qiagen) to identify molecular networks within the Ingenuity Knowledge Database. *P* values (threshold of 0.05) and z-scores were used to identify significant upstream regulators. *P* value indicated significance, while z-scores were used to define activation (z-score ≥ 2.0) or inhibition (z-score ≤ −2.0). Access to raw data concerning this study was submitted under Gene Expression Omnibus (GEO) accession number GSE149853.

### Statistical analysis

Numerical data are shown as a dot plot. Data are expressed as means ± SEM. Statistical significance between groups was determined using two-tailed *t* tests. One-way analysis of variance (ANOVA) was used for comparisons among three groups, while Kolmogorov–Smirnov test was used for comparisons between two groups. Fisher’s exact test was used to calculate *P* values in IPA upstream analysis. Statistical significance in survival data was determined using the log-rank test. All the statistical analyses (except for RNA-Seq data) were performed using GraphPad Prism 7 (GraphPad Software).

## Results

### Cytarabine treatment promotes transient AML cell motility reduction

To establish an AML syngeneic mouse model, we transplanted C1498 murine AML cells intravenously into wild-type C57BL/6 J mice [14, 15]. Prior to cell transplantation, C1498 cells were fluorescently labeled with GFP by retroviral transduction, allowing for tracking of the engrafted AML cells. The majority of the mice died between 25 and 30 days after AML cell transfer (Fig. S2); hence, we stratified the disease progression stages into early phase (7–13 days after transplantation), middle phase (14–20 days after transplantation), and late phase (day 21 until death).

**Fig. 1:**
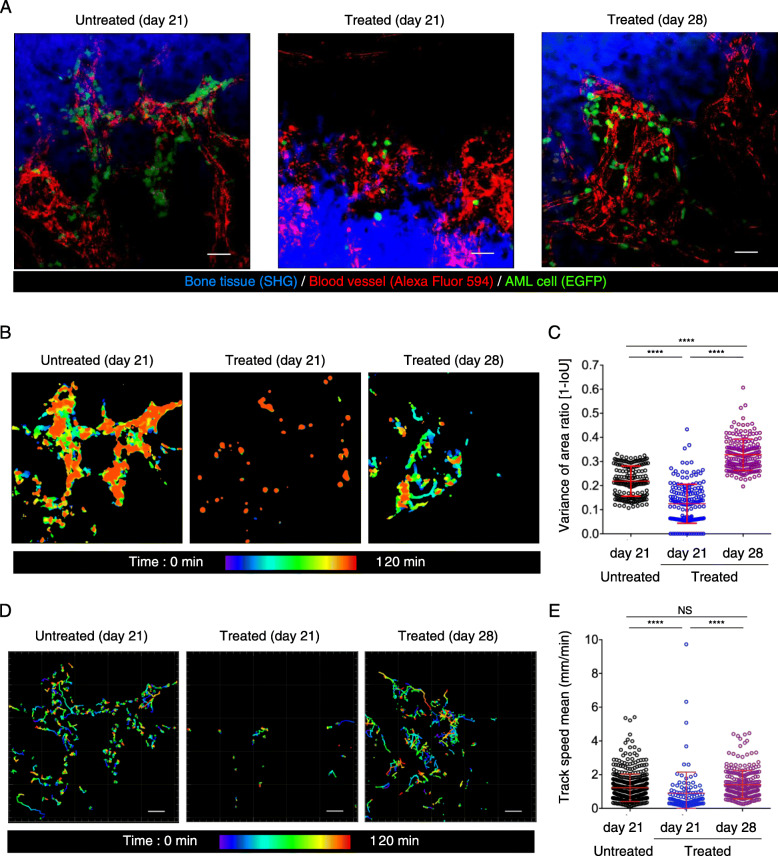
Dynamics of AML cells in bones during chemotherapy. **a** Representative intravital two-photon maximum-intensity projection (MIP) skull images at 21 and 28 days after AML cell transplantation. Images from untreated AML-transplanted mice at day 21, treated AML-transplanted mice at day 21 and treated AML-transplanted mice at day 28 are shown. Green, GFP-expressing AML cells; red, blood vessels (Alexa Fluor 594); blue, bone tissues (second harmonic generation; SHG). Scale bar, 50 μm. See also Video 2. **b** Representative images showing AML cell area. Scale bar, 50 μm. **c** Scatter plots showing displacement area ratio. Data are presented as mean ± SD; *n* = 3 mice per group; ****P < 0.0001 (one-way ANOVA). **d** Representative images of migrating AML cell trajectories. Scale bar, 50 μm. **e** Scatter plots showing the mean track speed of all cells analyzed in (**d**). Data from three mice per group from independent experiments are shown. Untreated (21 days), *n* = 461; treated (21 days), *n* = 108; treated (28 days), *n* = 243. Data are presented as mean ± SD. ****P < 0.0001; NS, not significant (one-way ANOVA)

Intravital imaging of the parietal BM revealed a constant movement of AML cells along the blood vessels during all disease progression stages (Fig. S1; Video 1). We hypothesized that the development of chemoresistance in AML cells is accompanied by changes in cell motility; thus, we analyzed the dynamics of chemoresistant AML cells in the BM following cytarabine treatment. We administrated high-dose cytarabine (20 μg in 200 μL PBS) twice at days 19 and 20 after AML cell transfer; high-dose cytarabine treatment significantly prolonged median survival, and the number of AML cells were significantly decreased (Fig. S2). Although the number of AML cells in the BM transiently decreased in response to cytarabine treatment at day 21, between days 21 and 28, the number of AML cells in the BM increased (Fig. 1a), suggesting AML cell growth.

**Additional file 1: Video 1.** Intravital time-lapse imaging during different phases of progression of AML. AML cells were transplanted into wild-type C57Bl6 mice. AML mice were subjected to intravital time-lapse BM imaging during different disease phases. Green, EGFP-expressing AML cells; red, blood vessels (Alexa Fluor 594); blue, bone tissues (SHG). Scale bar, 50 μm.

To compare the dynamics of AML cells between the cytoreductive and proliferative phases after cytarabine treatment, we performed intravital time-lapse imaging on days 21 and 28 (Video 2). AML cell migration is characterized by amoeboid movements, which are driven by repetitive cycles of protrusion and contraction. Thus, we classified AML migration into two types: a constantly changing cell shape characterized by protrusions or migration driven by extensive contraction and protrusion. To quantify the extent of cell shape alterations, we calculated the “Intersection over Union (IoU)” of AML cells using time-lapse imaging data (Fig. 1b). The value of “1 minus IoU” decreased during the cytoreductive phase (Fig. 1c), suggesting reduced protrusion activity. By contrast, protrusion activity increased during the proliferative phase (Fig. 1c). The centroid tracking of individual AML cells revealed that the mean speed of AML cells during the cytoreductive phase was < 1 μm/min, and was significantly lower compared with the mean speed of AML cells during the proliferative phase or the control group (> 1 μm/min; Fig. 1d, e). These results suggest that cytarabine treatment induces AML cell motility changes likely by modulating the contractile machinery of the cells.

**Additional file 2: Video 2.** AML cell motility during chemotherapy. AML cells were injected into wild-type C57Bl6 mice. At days 19 and 20, the mice were treated with PBS or cytarabine. Intravital time-lapse imaging experiments were performed on day 21 (PBS control and cytarabine-treated mice) or day 28 (cytarabine-treated mice). Green, EGFP-expressing AML cells; red, blood vessels (Alexa Fluor 594); blue, bone tissues (SHG). Scale bar, 50 μm.

### Chemotherapy does not affect AML cell localization in the BM

**Fig. 2:**
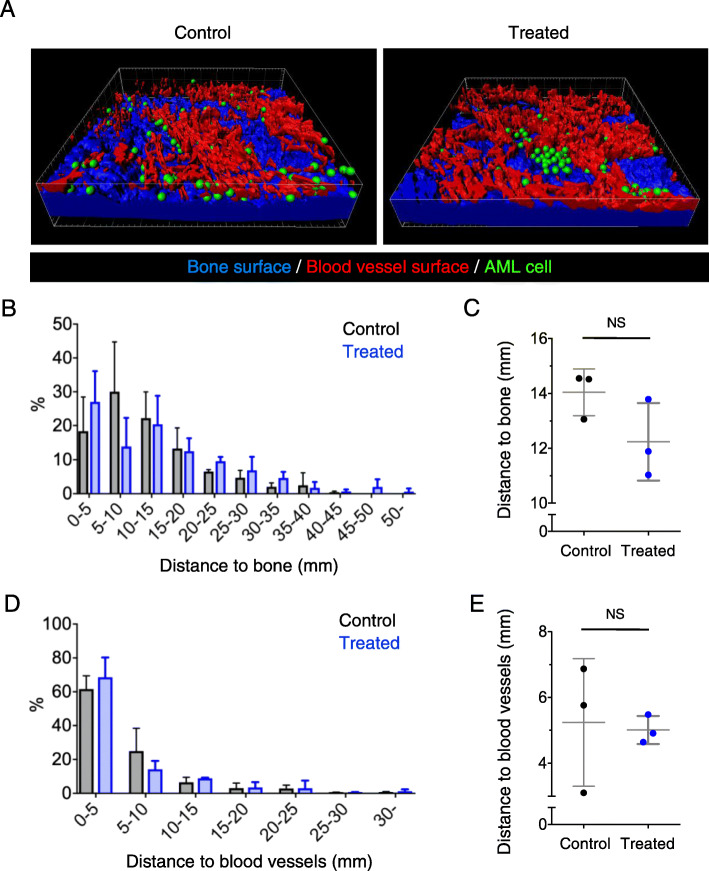
Localization of chemoresistant AML cells in the bone. **a** Representative intravital two-photon 3D skull images of AML control mice and AML mice treated with chemotherapy. Green spots, AML cells; red, surface of blood vessels; blue, surface of bone tissues. See also Video 2. **b**, **d** Distribution of distance between AML cells and the bone surface (**b**) or blood vessels (**d**). Pooled data from three mice per group are shown. Control, *n* = 400; treated, *n* = 235. **c**, **e** Mean distance between AML cells and the bone surface (**c**) or blood vessels (**e**). NS, not significant (Kolmogorov–Smirnov test)

Although AML chemoresistance has been reported to rely on the interaction with components of the BM microenvironment, it remains unclear whether AML cell localization within the BM can induce chemoresistance. To determine the localization of chemoresistant AML cells within the BM, we performed three-dimensional (3D) imaging using two-photon microscopy using mice injected with AML cells and subsequently treated with high-dose cytarabine at days 19 and 20 after AML cell transfer. After constructing the pseudo-surface of blood vessels and bones using the Imaris software, we calculated the distance from each AML cell to the nearest blood vessel and bone surface (Fig. 2a). More than 50% of AML cells were localized within 15 μm of bone surfaces in untreated control and cytarabine-treated mice (Fig. 2b, c). Similarly, more than 60% of AML cells were localized within 5 μm of blood vessel surfaces in control and cytarabine-treated mice (Fig. 2d, e). These results suggest that there is no association between AML cell chemoresistance and cell localization in the BM.

### Inhibition of MRTF-SRF signaling reverses chemoresistance and cell motility changes in AML cells

**Fig. 3:**
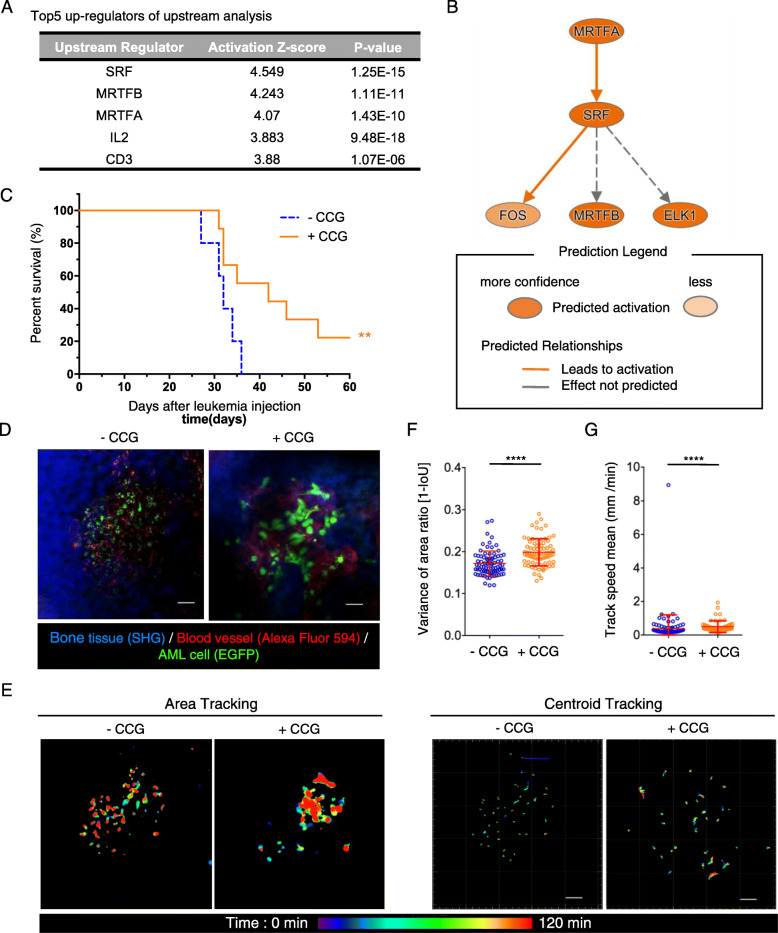
The effect of CCG treatment during chemotherapy on AML cells. **a** Predicted activity of the top five upstream regulators based on the gene expression profiles of AML cells isolated from cytarabine-treated and untreated mice (21 days after AML cell transplantation). **b** Network analysis of the upstream regulators of MRTFA and its target genes. MRTFA activation led to SRF and MRTFB upregulation. **c** Kaplan–Meier plot showing survival of AML-injected mice treated with 200 μg CCG in 50 μL DMSO in addition to 20 μg cytarabine in 200 μL PBS. −CCG, *n* = 5; +CCG, *n* = 9; data from two experiments; **P < 0.0332 (log-rank test). **d** Representative intravital two-photon MIP skull images of AML control mice and AML CCG-treated mice. Green, EGFP-expressing AML cells; red, blood vessels (Alexa Fluor 594); blue, bone tissues (SHG). Scale bar, 50 μm. See also Video 3. **e** Representative images showing displacement area (left) and trajectories (right) of AML cells analyzed in (**d**). Scale bar, 50 μm. **f**, **g** Scatter plots showing displacement area ratio (**f**) and mean track speed (**g**) of all analyzed cells. The data were pooled from three or four mice per condition from independent experiments. −CCG, *n* = 80 (**f**), *n* = 119 (**g**); +CCG, *n* = 80 (**f**), *n* = 54 (**g**). Data are presented as mean ± SD. ****P < 0.0001 (Kolmogorov–Smirnov test)

To further characterize the chemoresistant AML cells, we analyzed the gene expression profiles of AML cells from cytarabine-treated mice 21 days after AML cell transfer by RNA sequence. Enrichment analysis for diseases and biological functions using the Ingenuity Pathway Analysis (IPA) software revealed that the biological functional categories “cellular movement” and “immune cell trafficking” were significantly suppressed in cytarabine-treated AML cells (Fig. S3A, B). This transcriptional profile was consistent with our intravital imaging results (Video 2). To identify upstream regulators that determine AML cell chemoresistance, we conducted upstream analysis and causal network analysis using IPA. Serum response factor (SRF) and the myocardin-related transcription factors MRTFA (MKL1) and MRTFB (MKL2) were the top three upstream regulators (Fig. 3a). Using IPA network analysis, we found that extensive interactions exist between these three regulators (Fig. 3b). Additionally, DIAP is the actin nucleation and elongation factor that drives serum-dependent SRF-MRTFA, as well as several Rho guanine nucleotide exchange factors, were identified as active master regulators in cytarabine-treated AML cells (Fig. S3C). Recent studies showed that activation of MRTF and SRF is Rho-dependent. Moreover, MRTF and SRF have been implicated in cancer cell motility regulation by modulating the construction of actin filament networks [18–20].

To further investigate the role of Rho-MRTF-SRF pathway activation in AML cell motility following cytarabine treatment, we used CCG-203971 [21], a CCG-1423 analog that inhibits Rho-induced SRF-mediated transcription [22]. CCG-203971 (200 μg in 50 μL DMSO) was administered five times to cytarabine-treated mice. Kaplan–Meier survival analysis revealed that CCG treatment significantly prolonged survival (Fig. 3c), suggesting that Rho-MRTF-SRF inhibition augments the anti-tumor effects of cytarabine. We also assessed the effects of CCG treatment on the localization of chemoresistant AML cells using 3D imaging and surface distance analysis. We found that CCG treatment did not affect the distance between AML cells and blood vessels or bone surfaces (Fig. S4A–D). We also investigated the effect of CCG treatment on AML cell motility and found that CCG treatment promoted continuous cell morphology alterations (Fig. 3d, f; Video 3). The centroid tracking of individual AML cells revealed that the mean speed of AML cells was significantly higher in CCG-treated mice compared with control mice. However, the actual mean speed in CCG-treated mice was less than 1 μm/min, similar to that in the control group (Fig. 3e, g). Considering that the average diameter of AML cells is 10 μm, migration with a speed of less than 1 μm/min can be considered passive migration. Rapidly migrating AML cells were rarely observed in our imaging data (Video 3). Hence, Rho-MRTF-SRF inhibition following cytarabine treatment enhanced AML cell protrusion but not contraction. Considering the critical role of the MRTF-SRF pathway in the survival of chemoresistant AML cells, the combination of cytarabine with Rho-MRTF-SRF inhibitors could be a promising therapeutic approach for hematological malignancies.

**Additional file 3: Video 3.** CCG treatment induces dynamic changes in chemoresistant AML cells. AML-transplanted mice were treated with CCG before/during chemotherapy and after chemotherapy, followed by intravital time-lapse BM imaging. Green, EGFP-expressing AML cells; red, blood vessels (Alexa Fluor 594); blue, bone tissues (SHG). Scale bar, 50 μm.

## Discussion

Using intravital BM imaging, we showed that high-dose cytarabine treatment suppressed the migration of C1498 murine AML cells, consistent with a previous study using an MLL-AF9 AML mouse model [7]. Additionally, we demonstrated that proliferative C1498 AML cells regain their migration abilities after chemotherapy. Reduction of cell motility in residual AML cells upon chemotherapy could be explained by two ways: (i) chemotherapy reduced the motility of residual AML cells, and (ii) less motile AML cells were chemoresistant and could survive. Although we could not exclude the possibility of the latter case (ii), we think the former explanation (i) would be more likely because the number of “less motile” residual cells after chemotherapy was much larger than that of less motile cells inherently in control conditions. Several studies suggested an important role of the BM microenvironment in the development of chemoresistance in AML [6, 12, 23, 24]. Consistently, our findings suggest that extensive interactions occur between AML cells and BM microenvironment components during chemotherapy, which can promote the development of chemoresistance in residual AML cells. Osteoblasts, endothelial cells, and stromal cells are among the cell types in the BM niche that have been demonstrated to contribute to the development of chemoresistance in AML [25]. Nevertheless, a recent study involving in vivo imaging of the BM reported that the role of stromal cells in AML chemoresistance was negligible [7]. In this study, we found that the localization of AML cells within the BM did not influence the development of chemoresistance. Hence, the role of osteoblasts, endothelial cells, and stromal cells in the development of chemoresistance in AML remains unclear. Future studies involving the simultaneous tracking of various cell types are required to dissect the complex interplay between the components of the BM microenvironment and AML cells in vivo.

## Conclusions

Here, we demonstrated that the Rho-MRTF-SRF signaling pathway was strongly upregulated in chemoresistant AML cells. Rho-MRTF-SRF pathway activation enhances cancer cell motility in solid tumors, leading to cancer cell migration and metastasis. MRTF-SRF pathway inhibition using CCG-1423 or its derivative CCG-203971 exerted anti-cancer effects in metastatic malignant melanoma and breast cancer [19, 21]. Our imaging and transcriptomic findings showed that, in contrast to solid tumors, chemotherapy suppressed AML cell migration, as well as provided novel insight into the role of the Rho-MRTF-SRF pathway in the migration of chemoresistant AML cells. Nevertheless, similar to solid tumors, Rho-MRTF-SRF inhibition by CCG treatment augmented the anti-tumor effects of chemotherapy in our AML mouse model. Our findings on the relationship between cell motility and AML chemoresistance provide a strong rationale for the modulation of cellular motility as a therapeutic target for refractory AML.

## Supplementary information

**Additional file 4: Supplementary Figure 1.** Intravital two-photon imaging at different phases of progression. Representative intravital two-photon MIP skull images from C57Bl6 mice 10 days (left), 18 days (middle), and 27 days (right) after AML cell injection (day 0). Green, EGFP-expressing AML cells; red, blood vessels (Alexa Fluor 594); blue, bone tissues (SHG). Scale bar, 50 μm. See also Video 1.

**Additional file 5: Supplementary Figure 2.** Survival of cytarabine-treated AML-recipient mice. (A) Kaplan–Meier plot showing survival of AML-injected mice treated with PBS or 200 mg/kg of cytarabine. (PBS, n = 5; cytarabine, n = 5, from a single experiment). **P < 0.0332 (log-rank test). (B) Scatter plots showing the cell number of AML cells included in 10,000 of BM cells. Data are presented as mean ± SD. *P < 0.05 (Kolmogorov–Smirnov test).

**Additional file 6: Supplementary Figure 3.** Activation of SRF-MRTFA pathway after chemotherapy. (A) Top biological functions of the differentially expressed genes between chemotherapy-treated and control AML-recipient mice. (B) Heatmap showing differential expression genes after cytarabine treatment, as well as diseases and biological functions they are involved in. (C) Top five master regulators determined by causal network analysis.

**Additional file 7: Supplementary Figure 4.** Localization of AML cells in the BM after CCG treatment. (A, C) Distribution of distance between AML cells and the bone surface (B) or blood vessels (D) after CCG treatment. Pooled data from three mice per condition from independent experiments are shown. -CCG, n = 250; +CCG, n = 130. (B, D) Mean distance between AML cells and the bone surface (B) or blood vessels (D). NS, not significant (Kolmogorov–Smirnov test).

## Data Availability

The authors confirm that the data supporting the findings of this study are available within the article or its supplementary materials. Raw data were generated at Osaka University. Access to raw data concerning this study was submitted under Gene Expression Omnibus (GEO) accession number GSE149853. Derived data supporting the findings of this study are available from the corresponding author E.Y. and M.I. on request.

## Abbreviations

AML: Acute myeloid leukemia
ANOVA: Analysis of variance
BM: Bone marrow
FBS: Fetal bovine serum
GEO: Gene expression omnibus
IPA: Ingenuity pathway analysis
IoU: Intersection over union
MIP: Maximum intensity projections
MKL: Megakaryoblastic leukemia
MRTF: Myocardin-related transcription factors
PBS: Phosphate-buffered saline
SHG: Second-harmonic generation
SRF: Serum response factor
