# Supplementary material for: Migration arrest of chemoresistant leukemia cells mediated by MRTF-SRF pathway
Source: Inflamm Regen. 2020 Jul 6;40:15. doi: 10.1186/s41232-020-00127-6 (PMC7336645; doi:10.1186/s41232-020-00127-6)
Supplement: Supplementary file 1 [file 41232_2020_127_MOESM4_ESM.docx]

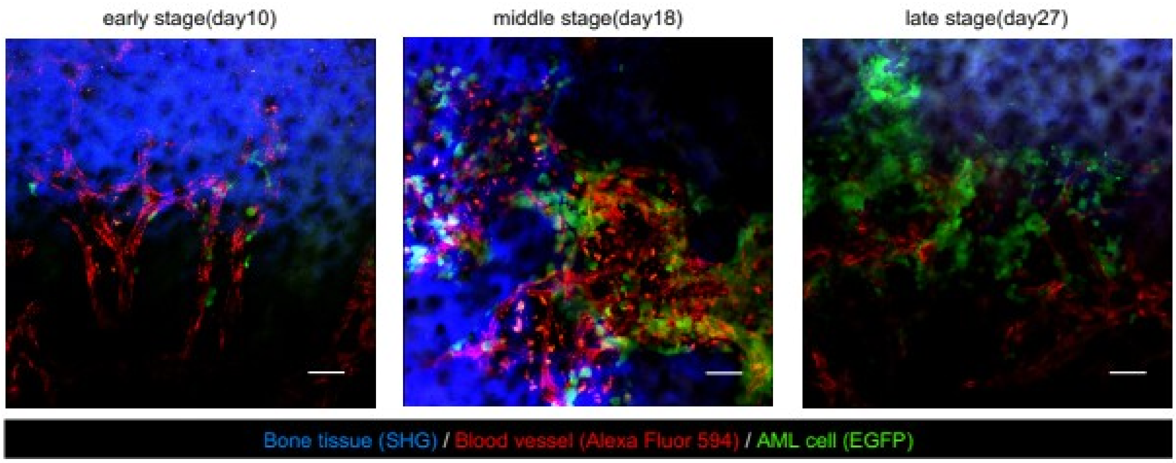
early stage(day10)
middle stage(day18)
late stage(day27)
Bone tissue (SHG) / Blood vessel (Alexa Fluor 594) / AML cell (EGFP)
