# Supplementary material for: Migration arrest of chemoresistant leukemia cells mediated by MRTF-SRF pathway
Source: Inflamm Regen. 2020 Jul 6;40:15. doi: 10.1186/s41232-020-00127-6 (PMC7336645; doi:10.1186/s41232-020-00127-6)
Supplement: Supplementary file 2 [file 41232_2020_127_MOESM5_ESM.docx]

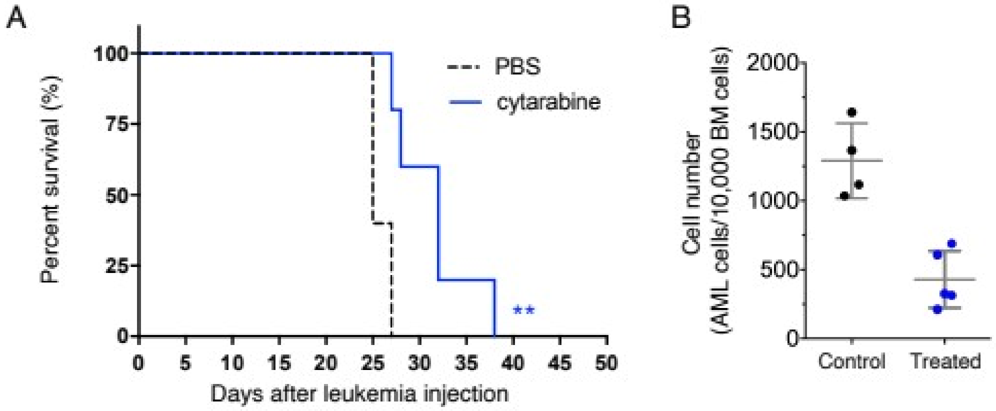
A
Percent survival (%)
100
75
50
25
0
PBS
cytarabine
**
0 5 10 15 20 25 30 35 40 45 50
Days after leukemia injection
B
Cell number
(AML cells/10,000 BM cells)
2000
1500
1000
500
0
Control
Treated
