# Supplementary material for: Migration arrest of chemoresistant leukemia cells mediated by MRTF-SRF pathway
Source: Inflamm Regen. 2020 Jul 6;40:15. doi: 10.1186/s41232-020-00127-6 (PMC7336645; doi:10.1186/s41232-020-00127-6)
Supplement: Supplementary file 3 [file 41232_2020_127_MOESM6_ESM.docx]

A Top Diseases and Biological functions

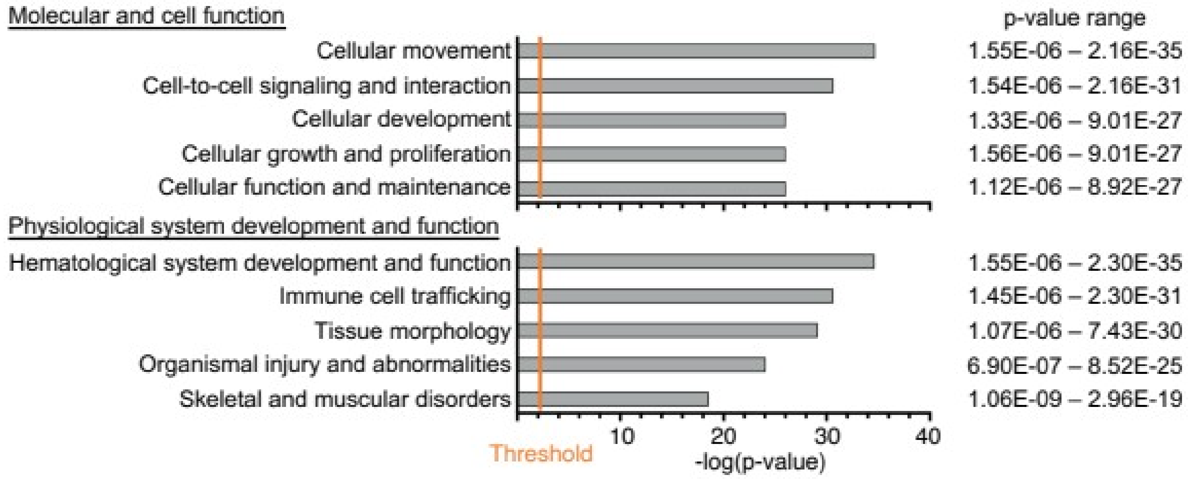

B

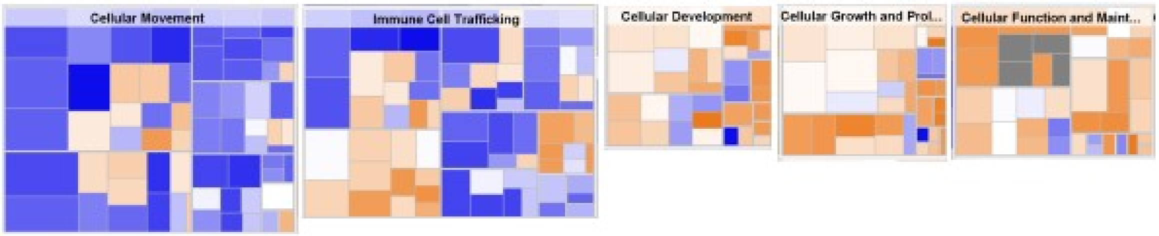

C Top5 active master regulators of causal network

Master Regulator	Participating regulators	Z-score	P-value
TNFRSF12A	NFkB (complex), RHOA, SRF, TNFRSF12A	5.252	1.09E-20
Diap	Akt, APC, Diap, DIAPH1, DIAPH3, DLG1, ERK1/2, FOS, GSK3B, ITGAM, MAPK1, MAPK14, MAPK3, MAPK8, Nfat (family), NFATC1, NFkB (complex), P38 MAPK,PAX6,PTK2,RUNX2,SMAD3,SRF	5.06	5.24E-19
MCF2	CDC42, IKBKB, Jnk, JUN, MAPK14, MAPK8, MCF2, NFkB (complex), Ras homolog, RHOA, RPS6KB1, Sapk, SRF	5.047	1.23E-21
ARHGEF2	ARHGEF2, JUN, MAPK14, MAPK8, NFkB (complex), RAC1, RHOA, SRF	4.95	4.07E-22
ETS-ELK1	ELF1,ELF3,ELF4,ELK1,ETS,ETS-ELK1,ETS1,ETS2,FOS, Histone h3,MAPK3,SRF	4.808	3.72E-16
