# Supplementary material for: Migration arrest of chemoresistant leukemia cells mediated by MRTF-SRF pathway
Source: Inflamm Regen. 2020 Jul 6;40:15. doi: 10.1186/s41232-020-00127-6 (PMC7336645; doi:10.1186/s41232-020-00127-6)
Supplement: Supplementary file 4 [file 41232_2020_127_MOESM7_ESM.docx]

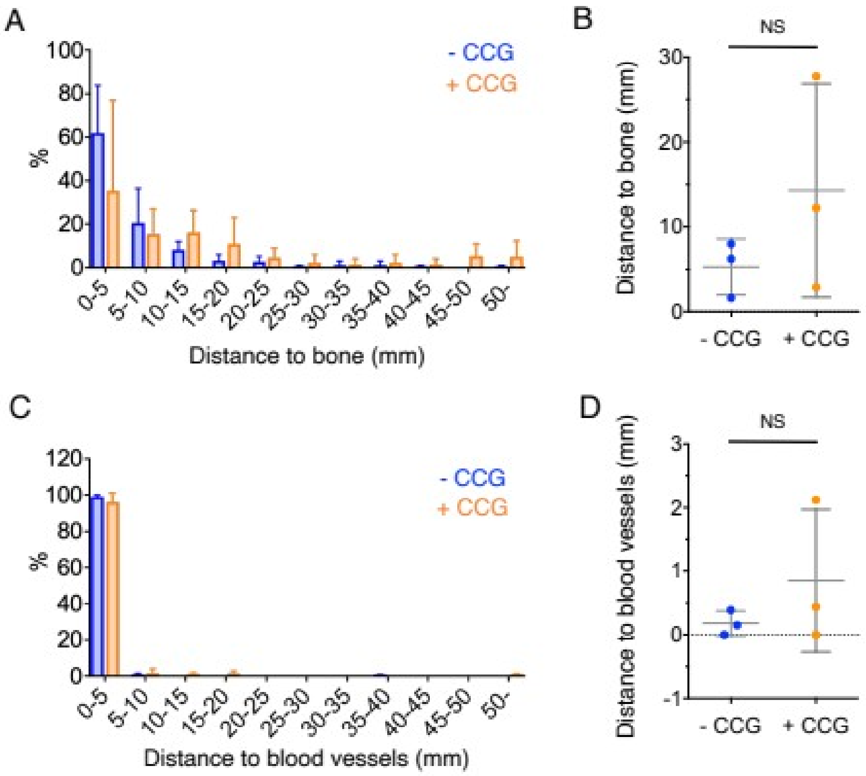

A
- CCG
+ CCG
%
0-5
5-10
10-15
15-20
20-25
25-30
30-35
35-40
40-45
45-50
50-
Distance to bone (mm)
B
NS
Distance to bone (mm)
- CCG
+ CCG
C
- CCG
+ CCG
%
Distance to blood vessels (mm)
D
NS
Distance to blood vessels (mm)
- CCG
+ CCG
